# Welfare Assessment following Heterotopic or Orthotopic Inoculation of Bladder Cancer in C57BL/6 Mice

**DOI:** 10.1371/journal.pone.0158390

**Published:** 2016-07-27

**Authors:** Amy Miller, Hannah Burson, Ariane Söling, Johnny Roughan

**Affiliations:** 1 Institute of Neuroscience, Comparative Biology Centre, Newcastle University, Newcastle upon Tyne, NE2 4HH, England; 2 Research Institute of Pharmaceutical Sciences and Departments of Psychology and Pharmacology, Peabody Building, University of Mississippi, Oxford, MS, 38677, United States of America; 3 MDKN, Wilamowitzweg 11, 37085, Göttingen, Germany; Harvard University Faculty of Arts and Sciences, UNITED STATES

## Abstract

Few studies have assessed whether mice used as cancer models experience pain. Despite this possibility, the usual practice is to withhold analgesics as these are generally viewed as confounding. However, pain also alters cancer progression, so preventing it might not only be beneficial to welfare but also to study validity. Establishing the extent to which different cancer models result in pain is an important first step towards their refinement. We used conditioned place preference (CPP) testing and body-weight and behaviour analyses to evaluate the assumption that heterotopically implanted tumours result in less pain and fewer welfare concerns than those implanted orthotopically. C57Bl/6 mice received MB49^Luc^ luciferase expressing bladder cancer cells or saline implanted subcutaneously or into the bladder. These tumour-bearing or control groups underwent 2 daily 45 minute conditioning trials to saline or morphine (2mg/kg) and then a 15 minute drug-free preference test on day 3 of a 3 day cycle, continuing until the study ended. Tumours were imaged and behaviour data obtained following preference tests. Development of preference for the morphine-paired chamber (morphine-seeking) was determined over time. Heterotopic tumour development had no effect on morphine-seeking, and although the restraint used for heterotopic inoculation caused greater initial weight losses than anaesthesia, these mice steadily gained weight and behaved comparatively normally throughout the study. Orthotopic tumour inoculation caused no initial weight losses, but over the final 7 days these mice became less active and lost more body weight than cancer-free controls. This indicated orthotopic implantation probably caused a more negative impact on welfare or conceivably pain; but only according to the current test methods. Pain could not be confirmed because morphine-seeking in the tumour-bearing groups was similar to that seen in controls. Imaging was not found to be an effective method of monitoring tumour development surpassing manual tumour inspection.

## Introduction

With 50% of people now likely to experience cancer at some point in their lifetime [1] it is not surprising that the numbers of mice used in cancer research has risen to over 400,000 annually in the UK. Although many of these animals might develop pain, analgesics are rarely used as they could confound results; for example by altering the baseline rate of tumour development. Whereas non-steroidal anti-inflammatory drugs (NSAIDs) generally suppress tumour growth [2–4], depending on the drug chosen and model type, opioids can either promote or suppress various pathways involved in tumour progression [5–7]. Cancer researchers have to make vital decisions as to how to minimise such confounds in order to maximise model validity and the translational potential of findings. However, it can be forgotten that animals that are experiencing pain could just as easily provide incorrect results; hence in some cases it might be a better approach to try to prevent this. The route of cancer inoculation is another important issue in undertaking such cost-benefit analyses. Heterotopic models usually involve subcutaneous inoculation, and although these are sufficient for preliminary trials, with regard to translational value they are perceived to provide less relevant findings than orthotopic implants. This is because orthotopic implants are into the tissue(s) of origin and development occurs in an appropriate microenvironment; hence data on rates of angiogenesis, metastasis and the responses to therapy are considered more informative [8, 9]. Heterotopic models are generally viewed as more benign, so are seen to address researchers’ obligations to minimise suffering. However, few studies have been undertaken to specifically determine the relative impact on welfare of these different inoculation procedures. Monitoring aspects such as body weight, behaviour and changes in peripheral nociception can provide early indications of problems or evidence of pain [10, 11], but only indirectly. Developing effective monitoring tools has now become even more important with the advent of new legislation requiring retrospective severity assessment and more effective cost-benefit analyses (Directive 2010/63/EU). As a result, tests aimed at determining how pain ‘affects’ animals [12–14], such as the Conditioned Place Preference (CPP) procedure have grown more popular [15–19]. We have previously used this to show that C3H/HeN mice orthotopically implanted with bladder cancer progressively show a preference for a ‘place’ where they were exposed to morphine compared to one paired with saline. Crucially, this increased morphine-seeking not only exceeded the morphine preference of cancer-free controls, but was associated with heightened nociceptive responding and abnormal behaviour, and was most obvious in mice with larger tumours [20]. These data provided strong evidence of pain occurring up to 10 days before the study ended, and indicated a need for end-point refinement. The current study used a similar approach to gain evidence of whether pain might also arise in C57BL/6 mice during bladder cancer development, and as is widely assumed, if such welfare concerns are lessened if tumours are implanted heterotopically. Although there were more obvious impacts of orthotopic tumour development, for the reasons discussed, the need for end-point refinement in studies involving orthotopic implantation of bladder cancer in C57BL/6 mice remains uncertain.

## Materials and Methods

### Ethics Statement

All work was undertaken at the Comparative Biology Centre, Newcastle University, UK and adhered to the ethical and legal obligations of the Animals (Scientific Procedures) Act 1986 (UK Home Office Project/Personal license numbers; PPL 60/4431 and PIL 60/13195), EU Directive 2010/63 and the guidelines of the International Association for the Study of Pain (IASP). Final approval was from the Newcastle University Animal Welfare Review Body.

### Animals

To prevent additional animal use, 80 female C57BL/6J surplus animals were supplied by Charles River (Margate, Kent, UK). Forty were used in each study to match the design of our previous study [20] where a power calculation was performed to establish the appropriate numbers in each group. Mice in the heterotopic study were approximately 1–2 weeks younger (7–8 vs. 8–9 weeks old). Females were used to compare with our previous investigation and to reflect their more common use in bladder cancer studies due to the greater ease of urethral catheterisation. They were provided with food (R&M no.3 SDS Ltd, Whitham, UK) and tap water *ad-libitum*. They were singly housed in IVC cages (Type 1, (160mm (w) x 339mm (l) x 130mm (h)); Arrowmight, Hereford, UK) on sawdust bedding (Gold Chip, BS and S Ltd, Edinburgh, UK). ‘Sizzle nest’, a chew block and a cardboard tube provided enrichment (B&K Universal, Hull, UK). Acclimation was for 7 days in a holding room maintained at 23°C ± 1°C, 48% humidity, 15–20 air changes per hour and a 0700 to 1900 light cycle. Cages were cleaned weekly, retaining some soiled bedding to maintain home-cage familiarity. The enrichment materials were renewed as necessary. Once tumour development was confirmed (either via imaging or palpation) the mice were supplied with 2–3 soaked diet pellets left on the bedding. These were replenished each morning following weighing. Baseline weights were from the last day of the acclimation period, and after this the mice were weighed between 8 and 10am every morning.

### Tumour inoculation

The tumour cells were donated by Dr Ariane Söling (MDKN, Göttingen, Germany). They were cultured in Dulbecco’s Modified Eagle’s Medium (DMEM) supplemented with 10% foetal bovine serum, 1% penicillin/streptomycin, and 0.1% sodium pyruvate (Invitrogen, Paisley, Scotland). Assignment of mice for tumour implantation was based on the result of an initial CPP preference test (described below). Details of the orthotopic implantation procedure can be found elsewhere [20], but briefly, 2 batches of 20 mice were anaesthetised with isoflurane, and then half of the mice in each batch (i.e. 10) were implanted in the bladder with 5x10^6^ MB49^Luc^ cells in a solution of 50μl Dulbeccos’ Phosphate Buffered Saline (DPBS) using a 1ml syringe. The remaining 10 mice of the batch were implanted with the same volume of DPBS only. Syringes were secured to the tails with adhesive paper tape to leave the cells or DPBS *in-situ* for 60 minutes. After removing the syringes the mice recovered in an incubator set at 37°C for 30 minutes. The next batch of 20 mice was then anaesthetised and the implantation procedure was repeated.

In the heterotopic study the 20 mice assigned for tumour inoculation were restrained for subcutaneous (s/c) injection in the standard manner (http://www.procedureswithcare.org.uk/subcutaneous-injection-in-the-mouse). An area of fur about 2 x 2 cm was then shaved on the right flank midway between the fore and hind limbs before subcutaneously injecting 50μl of DPBS containing 2.5x10^6^ MB49^Luc^ cells with an insulin syringe. The remaining 20 mice were controls. These were shaved identically but injected with 50μl of DPBS. The orthotopically inoculated mice received double the number of tumour cells in normal anticipation of a fraction being voided in urine. Solutions were kept on ice but were warmed to body temperature before use. After inoculation the mice were returned to their home-cages.

### CPP Testing

**Fig 1:**
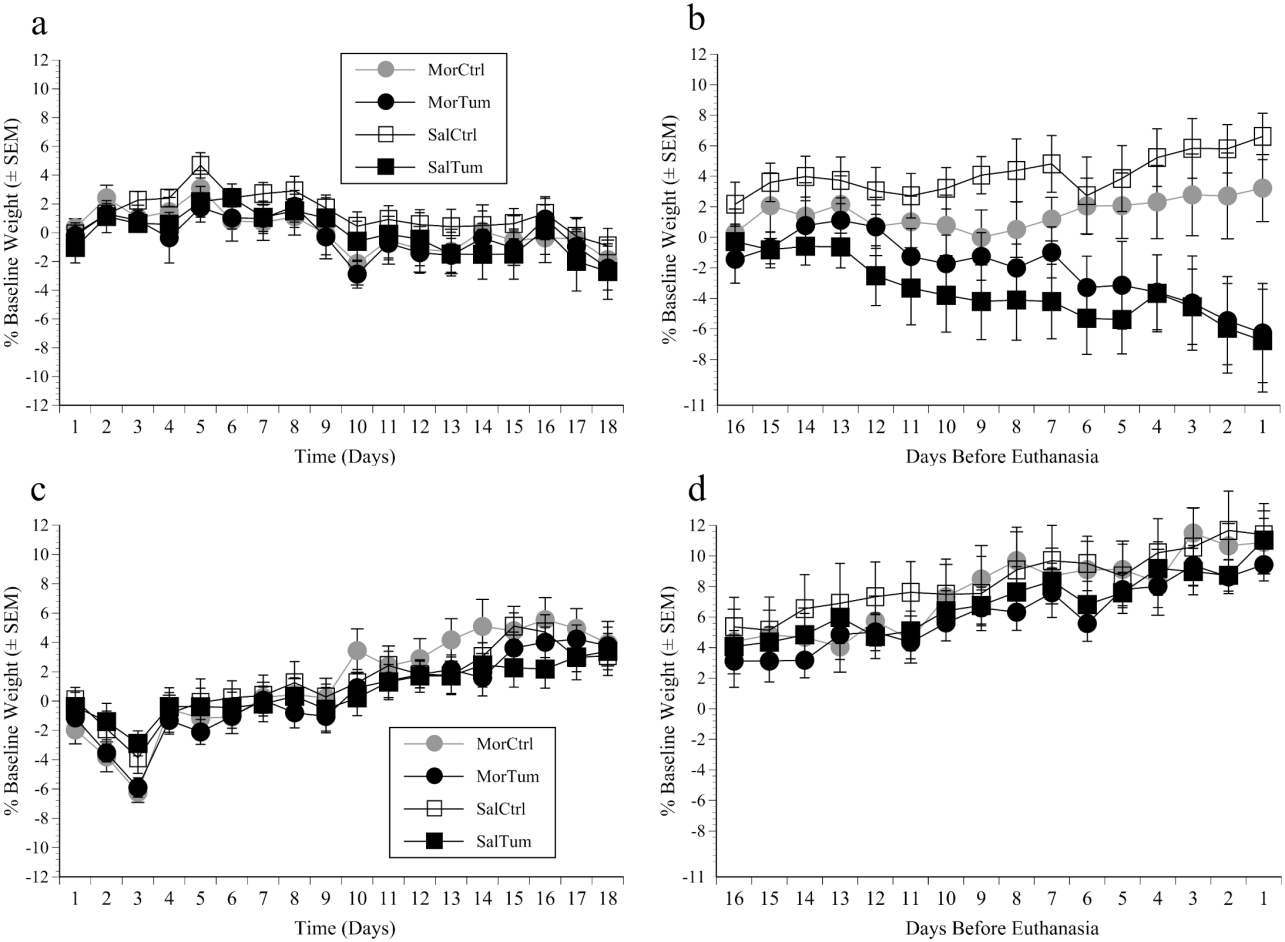
Body weight results. Mean percentage changes from baseline (pre-inoculation) body weight (±SEM) in mice inoculated with MB49^luc^ bladder cancer (Tum) or DPBS (Ctrl) and conditioned to morphine (Mor) or saline (Sal). Panels respectively illustrate data spanning the first 18 days (6 CPP Tests) and the final 6 CPP tests prior to euthanasia (16 days) in mice inoculated orthotopically (a, b) or heterotopically (c, d).

Six identical CPP test units were used (Model 3013AT, Med Associates, St Albans, VT, USA). Each unit had a black steel rod floored compartment (B) and a white steel grid floored compartment (W) separated by a grey solid-floored start box (G). The start box was separated from the B and W compartments by 2 programmable guillotine doors. Each compartment was equipped with an infra-red array to record chamber residence times and the total numbers of entries into and exits from each compartment. Initial preference testing was conducted one day prior to tumour inoculation. For this, mice were placed into the start box in ambient light for 1 minute following which the guillotine doors opened, the lights came on and the mice explored the apparatus for 15 minutes. Aside from during the 1 minute acclimation interval all chambers were equally illuminated at all times. The proportionate Black (B) or White (W) chamber preference (Pref) was then calculated (e.g. BPref = tB/(tB+tW); where t = total chamber residence time). Mice were randomly assigned to either the cancer or control group and for saline or morphine conditioning in their least preferred chamber (S+), but ensuring the between-group average B vs. W assignment was as close to 0.5 as possible [20]. Conditioning began the day following implantation with 2 sessions each day for 2 days. A drug-free preference test was conducted the next day (day 3) using the same method as for initial preference testing. This 3-day cycle (2 conditioning days and then a preference test day) was repeated until the study ended and the mice were euthanased. Black or white chamber conditioning was to saline in the morning (S-) and then saline or morphine each afternoon (S+). Half of the control and half of the tumour mice therefore received saline under the S- and S+ conditions (10 mice each); and these acted as controls for the effect of morphine. Only giving morphine in the afternoon was essential as there would otherwise have been carry-over effects from each morning conditioning session. All injections were given s/c. Morphine (Morphine sulphate, 30mg/ml; NHS Supplies, UK) was diluted with water for injection and given at 2mg/kg (~0.03mls per mouse). Saline controls received 0.03mls of 0.9% saline. Four hours elapsed between morning S- and afternoon S+ sessions. Successive preference for the S+ chamber (on the third day of each cycle) was calculated as for initial preference testing. The conditioning and test procedure is depicted in Fig 1 of our previous article [20].

### Behaviour analysis

The mice were filmed for behaviour analysis immediately after preference testing (between 2 and 4pm). Recordings only began once tumour development was confirmed either via imaging or palpation. In the orthotopic investigation this was after 13 days, whereas in the heterotopic study the first tumour was detected on day 3. Mice were placed into one of 3 clear plastic cages (32cm x16cm x 13cm, Techniplast UK Ltd) containing only sawdust bedding. They were recorded for 10 minutes using a video camera (Sony DCR-HC96, Sony, Japan) fixed to a tripod and positioned 30cm from the cage front. Cages were wiped with 70% ethanol between recordings. The video footage was analysed using automated behavioural analysis software (HomeCageScan; Clever Systems Inc., VA, USA (HCS). Details on this can be found elsewhere [20–24].

### Imaging

After filming, the mice were returned to their home-cages and moved a short distance to a room housing an IVIS Spectrum 200 (Xenogen, USA). They were injected s/c with 150mg/kg D-Luciferin (PerkinElmer, UK) and placed back into their home-cages. Mice were imaged 12 minutes later in groups of 3 or 4 following induction of anaesthesia with 5% isoflurane in 2 litres/min oxygen. The results section describes how a 12 minute delay was confirmed as the time of peak bioluminescent signal intensity. They were placed in the IVIS machine in dorsal recumbency on a stage heated to 36°C and anaesthesia was maintained by face-mask delivery of 2% isoflurane in 1.5–2 litres/min oxygen. Open filter scans of the appropriate body region (bladder or flank) were acquired with subject depth set at 1.5cm and exposure time set automatically. After imaging the mice recovered in their home-cages. The cancer-bearing mice were imaged every 3 days along with a randomly selected representative number of controls (5 conditioned to morphine and 5 to saline).

### Monitoring and end-point determination

Mice were checked for signs of tumour growth during daily weighing. Tumours implanted heterotopically were measured according to their length and width using digital calipers (‘Absolute Digimatic’; Mitutoyo Ltd., Andover, UK). The orthotopically implanted tumours were assessed by an experienced technician (CH) by gentle palpation of the bladder region between the thumb and forefinger. General condition was assessed each day; e.g. if mice appeared lethargic or had any mobility issues. Mucous membranes were inspected for normal appearance, and the coat for any piloerection or lack of grooming or dehydration (fur pinch test). In the orthotopic study any hematuria was noted whilst palpating the bladder, or if there was blood stained fur or bedding. Mice were earmarked for euthanasia once tumours were estimated to be in excess of 1cm diameter, or if there was >15% body weight loss and/or if they showed at least 2 of the clinical symptoms described above. In the heterotopic study mice were euthanased once tumours were >12mm at their widest point, if they lost >15% body weight, or if either of these coincided with any obvious lack of mobility. The decision to euthanase was made by consulting with the chief area technician (CH), NACWO or facility veterinarian.

### Nociceptive testing

Mice deemed close to end-point underwent nociceptive testing (Hargreaves method [25]) to determine their final nociceptive status. The decision as to whether it was humane to undertake this testing was made by the experienced technician (CH) or attending veterinarian. This was only allowed if mice were still mobile and responsive when examined by handling. Although there was no plantar injury caused by tumour growth, thermal response thresholds can alter due to the phenomenon of referred hyperalgesia. This can be used as an indirect method of assessing underlying pain status as has previously been shown during tumour development in mice [26] and in visceral pain models in rats [27–29]. The test apparatus had 6 clear plastiglas enclosures (11cm x 17xm x 14cm) in which mice acclimated for 3–5 minutes. The plantar test was then applied (Ugo Basile, Italy: Model 37370) with a heat intensity of 280mW/cm^2^ (30s cut-off time). Three readings were obtained from each hind-paw when mice were stationary and not grooming, alternating between paws and allowing at least 2 minutes between successive ipsilateral recordings. Faeces and urine were removed with a damp paper towel as necessary. They then received 2mg/kg morphine subcutaneously and were returned to their home-cages for 20 minutes. The enclosures were cleaned with a damp paper towel and then the test procedure was repeated.

### Euthanasia and *post-mortem* assessment

Mice were euthanased by cervical dislocation either immediately after completing nociceptive data collection, or if it was an imaging day, they were imaged and then overdosed with isoflurane in the IVIS machine before cervical dislocation. Primary tumours were immediately removed, measured and weighed and then imaged both intact and after being dissected in half using the same settings as for whole body imaging. The carcasses were then imaged to identify any metastases. A general inspection was then carried out following which the major organs including the heart, lungs, liver and kidneys were removed and weighed.

### Data processing

In the orthotopic study data collection continued in all control mice until the last tumour bearing animal was euthanased; matching our previous CPP bladder study design in C3H mice [20]. With hindsight this was unnecessary, so to reduce the burden of testing to the animals in the heterotopic study a randomly selected treatment-matched control was euthanased alongside each tumour-bearing animal. Analyses focused on the periods of potentially greatest impact on welfare; following tumour inoculation and approaching euthanasia (i.e. when tumours were most developed). Before first tumour detection the available data were compared between studies directly. However, because mice were euthanased at different times during the period approaching euthanasia these data were processed as we have previously described [20]. This was by aligning data from tumour-bearing animals with those from the same elapsed study time in randomly selected treatment-matched controls. In both studies the first mouse was euthanased after 6 CPP tests. Since the first test was on day 3, this was a total of 18 days. The last 6 CPP test cycles required a total of 16 days, so data spanning these two phases were used to assess welfare changes. The CPP outputs were; the proportionate total S+ chamber residence times, explorations (breaks of the first chamber beam), entrances (beams broken beyond the first), activity (any chamber beam break) and movements (a change in beams broken). The behaviour data underwent an initial analysis to identify the activities changing most due to tumour progression. Further details on this can be found elsewhere [20]. Relative changes in baseline body weight were calculated and were adjusted for final tumour wet-weight. Rates of tumour development were calculated as final wet-weight/total days, and final tumour burden as a proportion of final body weight. In the heterotopic study, tumour growth results were also available from the caliper measurements. The Hargreaves data were assessed according to mean response latency over the 3 left or right paw readings, and also the overall mean latency before and following morphine.

### Statistical analysis

All data were analysed using SPSS statistics software (IBM; Version 22). They were tested for normality and homogeneity of variance before applying GLM repeated measures with ‘Time’ as a within-subjects factor and cancer vs. control and morphine vs. saline as between-groups factors. Analyses were applied to the data obtained over the first 18 or last 16 days (each spanning 6 CPP tests). Correlation analysis (Pearson’s ‘r’) was used to assess relationships between morphine seeking and the kidney, tumour and body weight data, and if there were links between these and final tumour burden. Paired samples t-tests were used to analyse the nociceptive response data with probability levels corrected for multiple comparisons (*Bonferroni*) as appropriate. All Figs show mean values ±1SEM whereas values shown in the text and S1 Table are means ±1SD. A copy of the original data can be found in S1 Data.

## Results

There was no significant difference in the total number of days mice were enrolled following heterotopic or orthotopic tumour implantation (40±9 vs. 35±10 days), and this was also unaffected by whether mice received morphine. Tumour inoculation failed in 3 orthotopically implanted mice; 2 assigned for morphine and 1 for saline conditioning. Data from these 3 mice were therefore excluded.

### Body weight

One mouse in the heterotopic study was 5g lighter at baseline than all others so was excluded. Otherwise there were no significant differences in baseline body weights across the four treatment groups in each study. However, the mice in the heterotopic study were 1 week younger and initially weighed less (18±0.2 vs. 23±0.3g; f(1, 78) = 176, p<0.001). In the orthotopic study there were no weight differences over the first 18 days regardless of whether mice received morphine or a tumour, however ‘Time’ was significant (F(15, 495) = 12.5, p<0.001). This was because weights generally increased over the first 5 days (F(1, 74) = 40, p = 0.001), and following this there were either gains or small losses (Fig 1a). In the heterotopic study ‘Time’ was also highly significant as mice lost weight over the first 3 days but then also steadily gained (F(15, 525) = 64.4, p<0.0001). However, those receiving morphine showed greater initial losses (day 1–3) and then more rapid gains than those conditioned to saline; significant ‘Time’ x ‘Morphine’ interaction (F(15, 525) = 2.1, p = 0.009). Gains were also less rapid if mice were tumour-bearing; ‘Time’ x ‘Tumour’ was significant (F(15, 525) = 4.4, p<0.001; Fig 1b). The same analysis was applied to data from the last 16 days. In the orthotopic study the controls gained whereas the tumour-bearing mice lost between 5 and 10% (significant ‘Time’ x ‘Tumour’ interaction; F(15, 525) = 9.5, p<0.001, Fig 1c), and this was unaffected by morphine. In the heterotopic study all mice showed steady weight gains; also without any effects of morphine (‘Time’ significant (F(15, 540) = 39, p<0.001; Fig 1d).

### CPP

**Fig 2:**
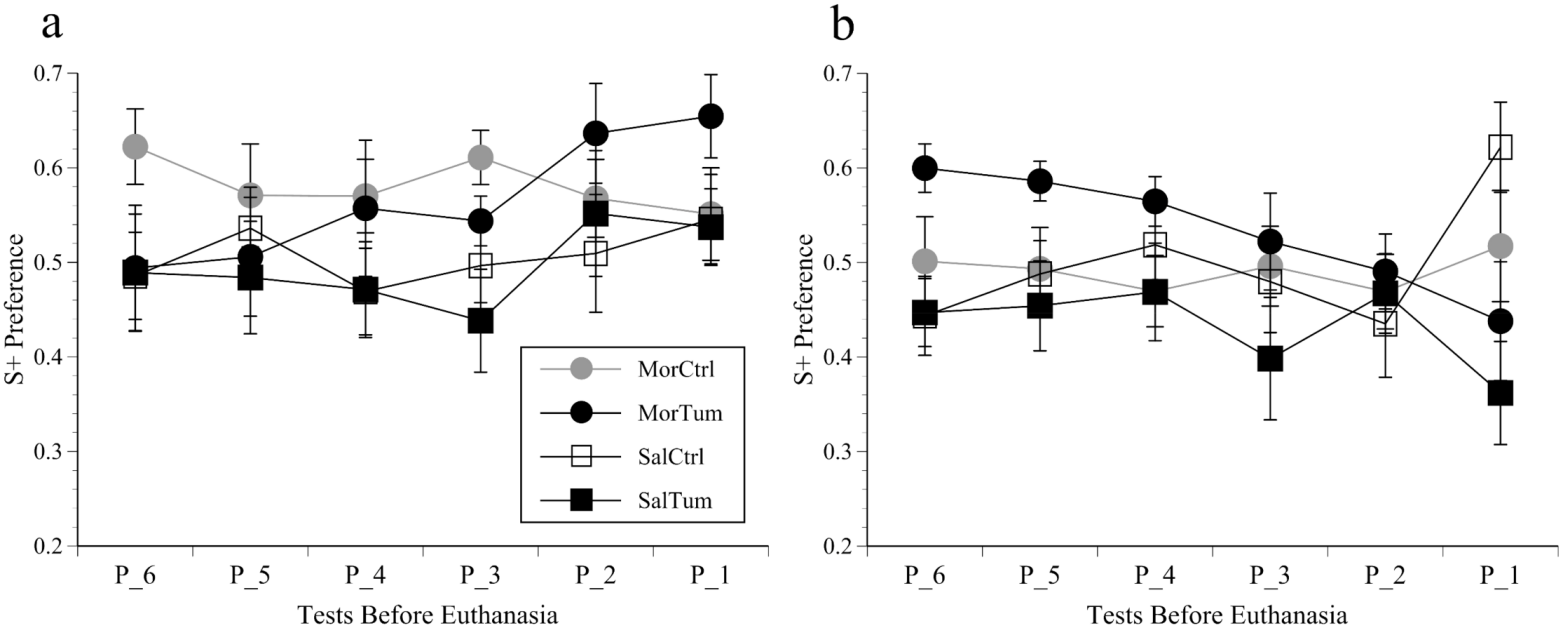
CPP results. Proportionate S+ chamber residence time (mean ±SEM) over the final 6 CPP cycles (P_6 to P_1; 16 days) before euthanasia in mice inoculated orthotopically (a) or heterotopically (b) with MB49^luc^ bladder cancer (Tum) or DPBS (Ctrl) and conditioned over a repeated 3 day cycle to morphine (Mor) or saline (Sal).

There were no significant CPP changes over the first 6 test cycles in either study depending on whether mice received morphine or were tumour-bearing. Over the last 6 tests (16 days) S+ chamber residence times increased in mice orthotopically implanted with tumours and conditioned to morphine, however, this was rendered non-significant as the same occurred over the last 2 tests in the saline-conditioned tumour group (Fig 2a). In the heterotopic study morphine had no overall effect; S+ scores increased over the last 2 tests in controls but reduced in the tumour groups (Fig 2b). Although both tumour groups showed a reduced S+ preference over these final 2 tests (‘Tumour’ factor significant (F(1, 37) = 13.6, p = 0.001)), this was not accompanied by increased time spent in the central grey zone. Had this been the case it might have indicated the mice had mobility issues. The other CPP measures (exploratory movements and entrance counts) were no more informative that total residence times so have not been depicted graphically.

### Behaviour

**Fig 3:**
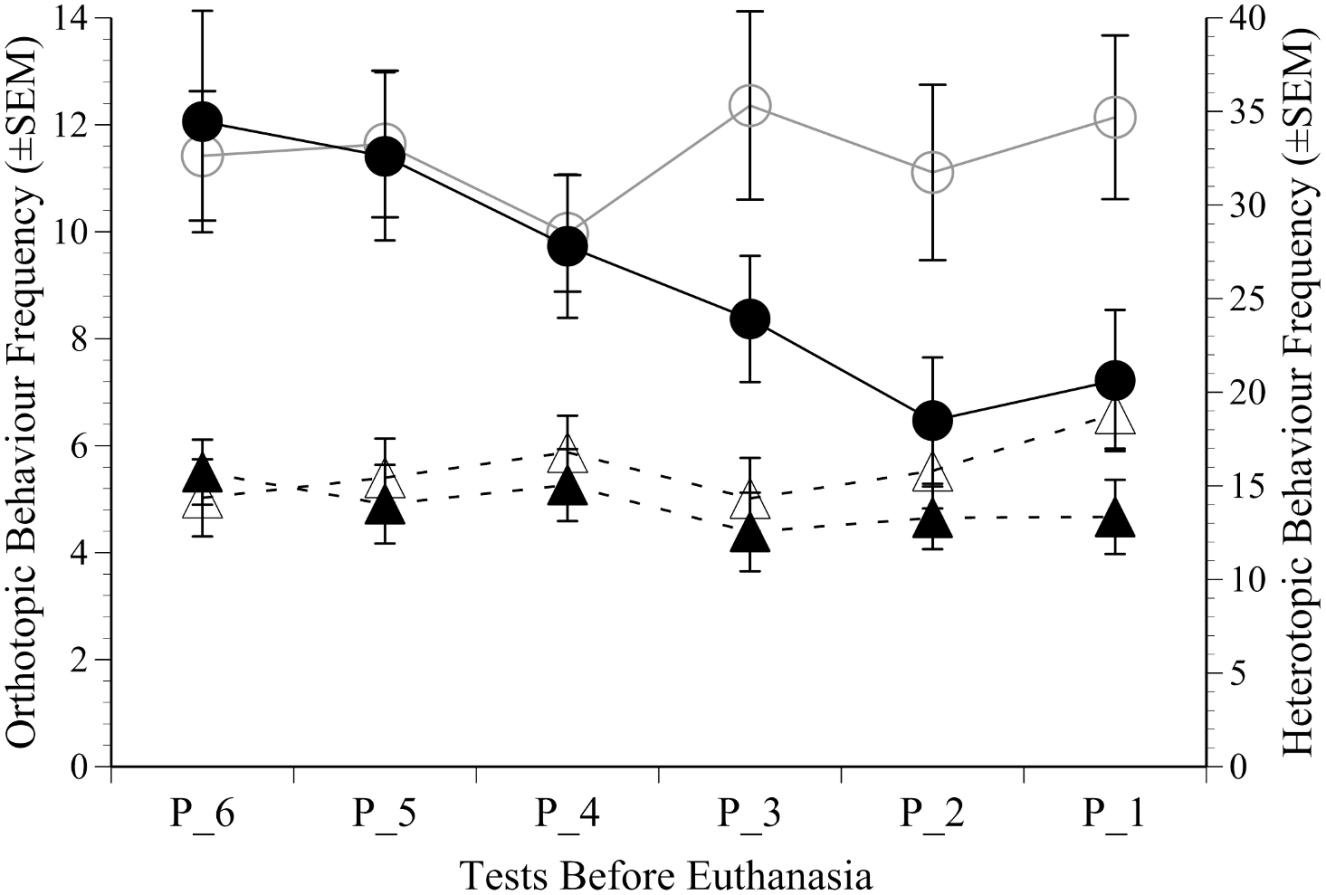
Behaviour results. The mean frequency of rearing (±SEM) in the orthotopic (left axis; circles) and heterotopic study (right axis; triangles) in tumour bearing and control groups (filled versus open symbols); showing the significantly greater decline in activity in the tumour-bearing mice in the orthotopic study from CPP test 3 (P_3) to just prior to euthanasia (P_1).

There were no significant behaviour alterations in either study prior to the last 16 days (6 CPP tests) and no effects of morphine. The only activities that were significantly impacted during this later phase of tumour development were low and high rearing. These were averaged and compared between groups. Heterotopic implantation caused no significant behaviour changes as the study approached end-point (Fig 3). Compared to controls the tumour-bearing mice implanted orthotopically progressively reared less over the last 3 CPP tests (7–10 days; ‘Time’ x ‘Tumour’; (F(5, 175) = 2.5, p = 0.031), although this was only a modest overall difference. There was no evidence indicating the anaesthesia sessions used for imaging had any influence on behaviour; i.e. the 10 non-tumour imaging controls behaved in the same way as their non-imaged counterparts.

### Tumour development

**Fig 4:**
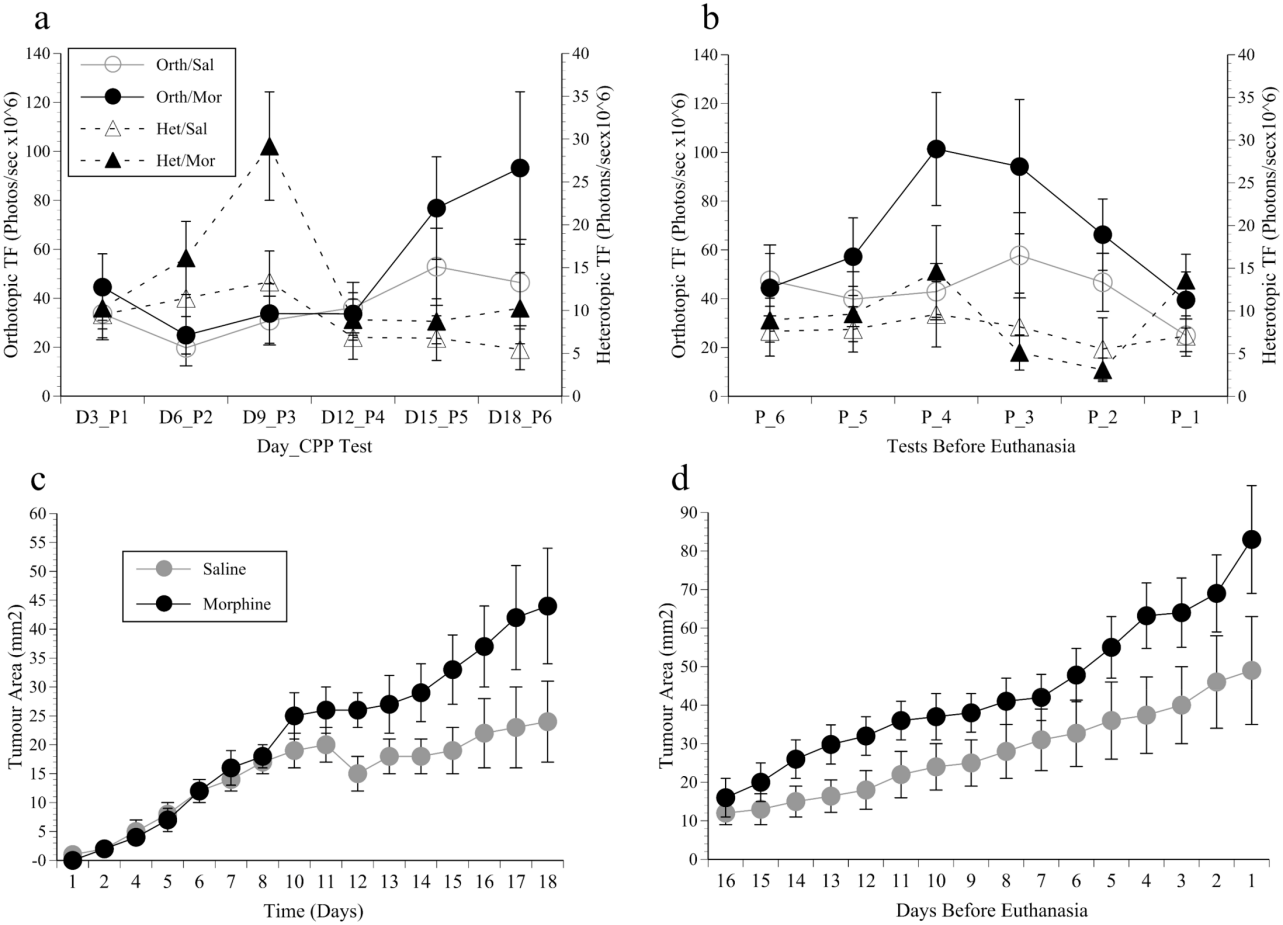
Imaging results. (a) the mean total flux (TF ±SEM) of bioluminescent signals emanating from tumours implanted orthotopically (Orth; left axis, circles) or heterotopically (Het; right axis, triangles) in mice conditioned to morphine (Mor; closed symbols) or saline (Sal; open symbols) and imaged every 3 days (beginning on day 3) for 18 days (Day_CPP Test); (b) Mean TF (±SEM) during the final 6 CPP tests (P_6 to P_1; 18 days) before euthanasia; (c) Caliper measurements showing the mean tumour surface area (mm^2^ ±SEM) of heterotopically implanted tumours over the first 18 post-inoculation days (6 CPP tests) and (d) last 16 days (6 CPP tests) in mice conditioned to morphine or saline.

Imaging was the main method of monitoring tumour growth, but in the heterotopic study tumours were also measured using calipers. Determination of the luciferin kinetic curve showed mean TF steadily increased until 12 minutes after luciferin administration, plateaued at 132±158 photons/second and then increased from 14 to 20 minutes to 148±176, but by then the signals were generally more variable. A 12 minute delay was therefore appropriate and was used in all cases. None of the controls showed any above-background signal at any time. Fig 4a shows the orthotopic and heterotopic imaging data from the first 6 CPP tests in the saline or morphine groups. Multiple comparisons were used to compare results between successive imaging days. In the orthotopic study bioluminescent signals were slow to develop, and it took until day 15 (CPP Test 5) before they were above that measured on day 6 (p = 0.007). After this they were highly variable, especially in the mice receiving morphine, and as a result the mean increase to day 18 was not significant. Heterotopic signal intensity increased from day 3 to 9 (F(1, 18) = 18.9, p<0.001), especially in mice receiving morphine (‘Time’ by ‘Morphine’ (F(5, 90) = 2.5, p = 0.037). This effect of morphine was most pronounced on imaging day 3 (elapsed day 9), but after this all signals declined dramatically. The results from the last 6 imaging sessions were also highly varied. The highest signal intensity was in the orthotopic study between tests 3 and 4 before euthanasia, which then significantly declined (‘Time’; F(5, 70) = 2.7, p = 0.027; Fig 4b). Although there was no overall effect of morphine, *post-hoc* analysis indicated enhanced signal intensity in the orthotopic mice receiving morphine spanning tests days 6 and 4 prior to euthanasia (F(1, 14) = 6.6, p = 0.022). The heterotopic results showed the same pattern; a modest signal increase from tests 6 to 4 followed by a significant decline towards the study end (‘Time’; F(5, 90) = 2.9, p = 0.016), but without any obviously greater intensity in mice given morphine. Fig 4c and 4d show the caliper–based estimates of heterotopic tumour size as the study progressed. Despite the apparent decline in TF in the imaging data, tumours steadily continued to develop over the first 18 days (‘Time’ significant (F(15, 270) = 22.78, p<0.001), and after day 12 tumours were clearly enhanced by morphine (CPP Test 4 onwards; (‘Time’ x ‘Morphine’; (F(15, 270) = 2.95, p<0.001); Fig 4c). The last 16 days (last 6 CPP/imaging cycles; Fig 4d) saw a significant increase in tumour growth until the study ended (‘Time’ significant; (F(15, 270) = 20.5, p<0.001), and although tumours were estimated as larger in the morphine group, not significantly. Haematuria was eventually observed in all orthotopically implanted mice with blood stained bedding and blood often being noted during palpation. This was first observed in one mouse on post-implantation day 11. Although this was not directly quantified it was used alongside the body-weight changes and the other clinical symptoms to inform end-point decisions.

### Nociceptive testing

Thermal responding was conducted on euthanasia days if possible. Paired *t*-tests indicated the mean of the 3 readings from the left and right paws was similar so the grand mean (left/right) was used in all analyses. The pre-morphine response latencies were similar between the tumour and control mice in both studies, and there was no evidence that this was affected by prior exposure to morphine (i.e. during conditioning). Morphine caused a general increase in nociceptive response latencies, but this was only significant in the orthotopic study (‘Time’ significant; (F(1, 31) = 9.8, p = 0.004). There was also no evidence of differentially lesser or greater responding depending on whether mice were implanted orthotopically or heterotopically.

### *Post-mortem* assessments

There were no metastases in either study and no obvious effects on any organ other than the kidneys in mice implanted orthotopically. The kidneys of these mice were significantly enlarged relative to controls (F(3, 36) = 3.7, p = 0.021) especially in mice with larger tumours (Pearsons’s R = 0.73, p = 0.001). Neither study showed a significant difference in final tumour wet-weights between the morphine or saline groups. The mean weight of orthotopically implanted tumours was 0.3±0.1g in both groups, and in the heterotopic study was 0.2±0.1g in the saline group and 0.4±0.1g in the morphine group. Rate of tumour development (weight/study days) and weight calculated as a proportion of final body weight also showed no significant overall effect of morphine. Final tumour wet-weights did not significantly correlate with either end-stage behaviour change or nociceptive status in either study.

## Discussion

Mice are an invaluable resource for developing treatments for human cancer, but are used at the potential cost of uncontrolled pain or generally poor welfare. Previously we used CPP testing and behaviour analysis to show C3H/HeN mice probably experience pain following orthotopic implantation of bladder cancer [20]. However, mouse strains are known to be differentially sensitive to pain [30], so in this investigation we attempted to establish if this also applies to C57BL/6 mice. We assessed two tumour inoculation methods predicting that orthotopically implanted tumours would more severely affect welfare; i.e. as these tumours grew there would be greater morphine-seeking and larger scale behavioural alterations and body-weight losses.

The main methodological difference from our previous study was that we used another non-metastasising bladder cancer cell line allowing tumours to be imaged. Compared to palpating tumours this was supposed to allow us to more precisely relate tumour growth stage to welfare status. However, the imaging results were generally unreliable as a means of monitoring development. For example, the first 9 days of the heterotopic study saw a particularly rapid signal increase in mice receiving morphine. Although this may have been due to an initial facilitation of luciferin uptake by tumours, and was a probable early indicator of the known pro-angiogenic effects of morphine [31], it was subsequently lost. The orthotopic signals were also variably affected by morphine; although at certain times they were increased, they were ultimately not indicative of eventual tumour burden assessed at autopsy. *Post-mortem* tumour imaging showed tumour necrosis was the most likely reason for this, and although there was no evidence this was affected by morphine, it has been found to be a problem with other luciferase-expressing orthotopic bladder cancers in mice [32], including the MB49 cell line [33]. Despite the imaging results, however, the *post-mortem* inspections confirmed tumours had continued to grow, and based on the heterotopic caliper estimates that they were indeed enhanced by morphine (Fig 4c and 4d). Although this was previously not found following orthotopic implantation of MBT-2 tumours, it may have been missed since at that time palpation was the only method of assessing tumour development. Although the mechanism by which opiates alter tumour growth is not fully understood, immune system suppression has most frequently been implicated where more rapid development occurs [34, 35]. The impact of morphine was not presently reflected in the tumour wet-weights, possibly because of desiccation during the more time-consuming process of removing and dissecting larger tumours.

The morphine dose rate was 2mg/kg throughout both investigations. This was based on our previous finding that at this dose it has no major rewarding effects but is still analgesic in C3H/HeN orthotopically implanted with a similar type of bladder cancer [20]. In that study cancer-related pain was indicated by increased morphine seeking coinciding with reduced active behaviour and body-weight over a similar study time-scale. Here, there was also an increased CPP to morphine as the orthotopic study ended, and the mice again lost body-weight and became less active. There was also the expected absence of, or reduction in morphine-seeking in cancer-free controls despite reducing the time of each CPP test from 45 to 15 minutes. In our previous study we hoped using 45 minute test cycles (but using only the first 15 minutes of data from each test) would add an essential extinction component. Then, by minimising the morphine preference of the controls we hoped to be able to more clearly distinguish any negatively reinforcing effects of morphine in the orthotopically implanted cancer group. However, this was subsequently not found to be helpful so preference tests were currently set to be 15 minutes only. An important study aim was to evaluate whether using the CPP approach might resolve some uncertainties as to the pain susceptibility of B6 mice. However, the current final preference of B6 mice for morphine in the orthotopic cancer group was more variable than previously found in C3H mice, and the total scores also averaged lower; reducing confidence that these end-stage effects were linked to pain in this strain also. Apart from our own previous work we know of only one other example where CPP testing has been applied to investigate the morphine preferences of B6 mice bearing orthotopically implanted tumours. Betourne et al. [36] found melanoma caused reduced morphine preference, and proposed that this was due to anti-opioid neuropeptide-related suppression of morphine reward. However, they used a substantially different study design to ours; a five-fold larger drug dose given on four occasions followed by a single preference test. Although it would be unwise to suggest a similar process occurred here, given the similarities between our previous orthotopic study in C3H mice it was still surprising that the B6 mice did not show similarly elevated morphine-seeking by the study end-point. Although B6 mice are sensitive to the positively reinforcing effects of morphine [37, 38], they have been found to respond more variably to different types of nociceptive stimuli than many other commonly used strains [30, 39]. It is possible, therefore, that in terms of ‘affect’ that they are either less or more variably reactive to the negatively reinforcing (i.e. pain-preventative) properties of morphine than some other strains.

With a view to informing future cost-benefit assessments the most important aim was to assess the effects of orthotopic relative to heterotopic tumour development in terms of impact on mouse welfare. We challenged the assumption that heterotopic models raise fewer concerns, such that in circumstances where translational potential is non-essential they are preferable to orthotopic models. Although the findings were not sufficient to say heterotopic implantation caused less pain, there were some obvious and at least one unexpected difference between the two models. The orthotopically implanted mice lost more weight as tumours grew and they became behaviorally less active; both of which are indicators previously taken as evidence of pain or another negatively affective state in mice [20, 40–42]. Orthotopic tumours also caused haematuria and enlarged kidneys, whereas in the heterotopic study, not only were there no major behaviour alterations but no end-stage weight losses. One might assume conditions causing hydronephrosis, including bladder cancer, would be painful. However, this is only described as painful by 26% of humans, and only if the cause has a rapid onset; e.g. the formation of renal calculi [43] which also causes obvious signs of pain in rats [29]. The most obvious conclusion from these collective findings is that orthotopic implantation, if not painful, was more stressful.

Hargreaves testing was intended to provide supplementary evidence of pain status such that mice with orthotopic tumours might become more nociceptive or differentially responsive to morphine. However, we observed only the typical opioid-induced increase in response latency without any model differences or relationship to eventual tumour burden; hence these data were less helpful than previously [20]. As is common, body-weight changes were used as a general indicator of welfare status. The restraint used for heterotopic tumour inoculation caused more initial losses, but once this weight was recovered these mice gained weight more rapidly; possibly because they were initially younger. However, offsetting these losses against an underlying more rapid growth rate means the initial effect of restraint may have been underestimated. Considering stress is a well-documented contributor to tumour growth variation, in terms of future study refinement anaesthesia may be more suitable than restraint as a means of immobilising animals for tumour implantation.

Although we have found no evidence that our present Home Office project severity banding of ‘moderate’ should be altered, it is still possible that individual mice, especially those with larger orthotopic tumours may have been more painful depending on the particular bladder or upstream region affected. This, therefore, does not point to any reduction in efforts to determine whether these and other mouse cancer models require refinement; for example by withdrawing animals earlier, or providing pain relief. The present use of morphine had some confounding effects, but with more uniform tumour development the Conditioned Place Preference testing procedure should still be a useful method of determining at what stage tumours affect welfare. In future studies we would therefore consider using an alternative drug for conditioning. Buprenorphine, for example, is both rewarding and analgesic in mice [41, 44], and in a recently completed study in BALB/c mice we have found it to be protective against surgical stress-induced proliferation of orthotopically implanted mammary carcinoma (unpublished results). As against this, it can increase metastatic colonization of the lungs so would be inadvisable for use in pain testing in immunocompromised mice [45]. Also, as with other assessment methods the value of the CPP approach towards welfare and end-point refinement may be highly strain dependent; given that van Loo et al., for example [46] found buprenorphine to have limited pain relieving properties in DBA/2 mice. We would not consider using NSAIDs since these generally impede tumour development [2–4], and because achieving effective pain relief is difficult even with excessively large dose rates of this class of drug [23, 40, 42, 47]. Although there could be disadvantages to using buprenorphine it at least seems to have fewer impacts on the immune system, which could explain why it has fewer overall effects on tumour development than some other opioids [5, 6].

Finally, it is important to say why the mice were housed singly and why they were female. Single housing the mice was not a decision taken lightly since these are social animals that are susceptible to isolation stress. This was done in an attempt to acclimate the mice to the environment they would be exposed to in the CPP apparatus, whilst collecting the behaviour data and during induction of anaesthesia before imaging. It also meant it was not necessary to perform ear-notching; which probably results in initial stress, and from which it is still not known how long mice need to recover. Using group-housed individually marked mice would have made it more difficult to select the correct individual from each cage at the appropriate time-point. This inevitably takes longer and requires additional handling and ‘cage-searching’, and may have significantly raised the chances of errors that could have virtually invalidated the results. Although they were alone, they were in semi-transparent cages adjacent to each other, consequently it was hoped any isolation stress would have been minimal. Females were used since apart from being an extension of our previous work [20] it is still not known how female mice differ from males in terms of sensitivity to circumstances eliciting poor welfare or pain [48]. Since there will inevitably be circumstances where female mice are necessary (e.g. studies of mammary carcinoma) these uncertainties can only persist if studies generally only ever use males.

## Supporting Information

S1 Table Study survival times. The mean number of days mice were enrolled (days ±SD) following orthotopic or heterotopic tumour implantation (Tum) whilst being conditioned to 2mg/kg morphine (Mor2) or Saline (Sal).
(DOCX)

S1 Data (XLSX)
