# Supplementary material for: Welfare Assessment following Heterotopic or Orthotopic Inoculation of Bladder Cancer in C57BL/6 Mice
Source: PLoS One. 2016 Jul 27;11(7):e0158390. doi: 10.1371/journal.pone.0158390 (PMC4962982; doi:10.1371/journal.pone.0158390)
Supplement: S1 Table — The mean number of days mice were enrolled (days ±SD) following orthotopic or heterotopic tumour implantation (Tum) whilst being conditioned to 2mg/kg morphine (Mor2) or Saline (Sal). (DOCX) [file pone.0158390.s001.docx]

| **Study** | **Heterotopic** | | **Orthotopic** | |
| --- | --- | --- | --- | --- |
| **Group** | Sal/Tum | Mor2/Tum | Sal/Tum | Mor2/Tum |
| **Days** | 36±9 | 33±11 | 42±9 | 41±9 |
